# Pure Iodide Multication Wide Bandgap Perovskites by Vacuum Deposition

**DOI:** 10.1021/acsmaterialslett.3c01094

**Published:** 2023-11-13

**Authors:** Isidora Susic, Lidón Gil-Escrig, Kassio P. S. Zanoni, Cristina Roldán-Carmona, Michele Sessolo, Henk J. Bolink

**Affiliations:** † Instituto de Ciencia Molecular, Universidad de Valencia, C/Catedrático J. Beltrán 2, Paterna, 46980 Spain

## Abstract

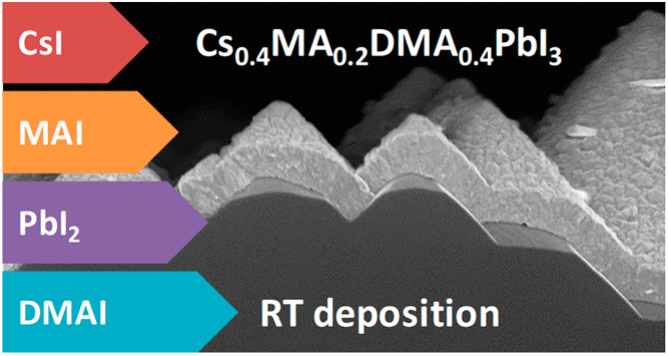

The CsPbI_3_ perovskite has a suitable bandgap (≈1.7 eV) for application in tandem solar cells. One challenge for this compound is that the semiconducting perovskite phase is not stable at room temperature, when it tends to form a yellow nonperovskite phase with a bandgap of approximately 2.8 eV. Therefore, many reports have been focused on the stabilization of the CsPbI_3_ black perovskite phase through the use of additives during solution processing. Vacuum deposited CsPbI_3_ has been seldom reported, as in this case, the insertion of stabilizing agents is more challenging. In this work, we demonstrate the vacuum processing of CsPbI_3_ perovskite films at room temperature, obtained by incorporating dimethylammonium iodide by cosublimation with CsI and PbI_2_. As-prepared films were applied in planar solar cells, leading to an average power conversion efficiency (PCE) exceeding 12%. In order to improve the device performance, we introduced a third A-site cation (methylammonium) in a four-source deposition process. This pure iodide formulation can be used in wide bandgap solar cells with a PCE up to 14.8%.

Metal halide perovskites are among the most promising materials in emerging photovoltaic technology, due to their tunable bandgaps, high absorption coefficient, long charge carrier diffusion length, and recombination lifetime.^1−5^ Perovskite solar cells with power conversion efficiency (PCE) close to 26% have been reached within a decade of development.^6^ The perovskite formulation in high efficiency devices is based on formamidinium lead iodide (FAPbI_3_), which is stabilized through additives to avoid the formation of a nonperovskite phase, which is otherwise more thermodynamically stable at room temperature (RT).^7^ Efficient wide bandgap perovskites are typically obtained using mixed iodide/bromide formulations,^8,9^ where mixed cations such as FA^+^ and Cs^+^ are employed to increase the phase stability and prevent (photoinduced) halide segregation.^10,11^ Chemically simpler alternatives are pure inorganic perovskites such as CsPbI_3_, with a wide bandgap (*E*_g_) of approximately 1.7 eV. However, the CsPbI_3_ perovskite phase is stable only at elevated temperature (>300 °C).^12,13^ In ambient conditions and at RT, CsPbI_3_ transitions to a yellow nonperovskite phase with *E*_g_ ≈ 2.8 eV, which is no longer interesting for photovoltaic (PV) applications.^14^ The origin of this instability can be understood by considering the Goldschmidt’s tolerance factor *t* = (*r*_A_ + *r*_B_)/, where *r*_A_, *r*_B_, and *r*_X_ are the ionic radii of A, B, and X in a general ABX_3_ perovskite, respectively. When 0.8 ≤ *t* ≤ 1, perovskites are generally stable, although in the lower part of this range, they may be distorted due to tilting of the BX_6_ octahedra and decreased symmetry. If *t* < 0.8, the A-cation is too small, destabilizing the perovskite phase and often leading to other alternative structures.^15^ The latter is the case of CsPbI_3_; therefore, many efforts have been focused on the stabilization of the CsPbI_3_ black perovskite phase by adjusting the tolerance factor via substitution/addition of larger A-site cations and/or smaller B- or X-site species.^16^ For example, the incorporation of molecules with larger ionic radius than Cs^+^ (*r* = 167 pm), such as methylammonium (MA^+^, *r* = 217 pm) and FA^+^ (*r* = 253 pm), can lead to stabilization of CsPbI_3_ at RT.^17,18^ In a similar fashion, incorporation of Br^–^ and Cl^–^ anions into CsPbI_3_ leads to better phase stability, although it typically results in wider bandgaps.^19−22^ Another widely explored strategy is the introduction of even bulkier organic cations, leading to the formation of quasi-2D and 2D layered perovskites.^23−25^ Dimethylammonium (DMA), with an ionic radius of 272 pm, has been successfully applied in the stabilization of the black perovskite phase of CsPbI_3_.^26−28^ It is worth noting that the stabilization of CsPbI_3_ using the intermediate known as hydrogen lead iodide (HPbI_3_), produced by adding HI in the solution, actually works via the formation of DMAI from the decomposition of dimethylformamide with HI, leading to a stable mixed A-cation Cs_1–*x*_DMA_*x*_PbI_3_ phase.^29^ There have been opposing reports of whether DMA is alloyed into the A-site or whether it has the role of an additive in a crystallization process. Wang et al. demonstrate that the role of DMAI is a crystal growth additive achieving a record PCE of 18.4%, and up to 19.0% with additional phenyltrimethylammonium chloride passivation.^30^ Marshall et al. revealed that DMA can be successfully incorporated into the CsPbI_3_ perovskite to form an alloyed composition of Cs_*x*_DMA_1–*x*_PbI_3_, which is more stable under atmospheric conditions than the original CsPbI_3_.^26^ The majority of these reports are based on solution-processing techniques (mainly spin coating), while vacuum deposition methods to prepare such stabilized CsPbX_3_ compositions have been scarcely investigated. Vacuum deposition offers several advantages, such as accurate control over the film thickness and composition, the possibility to fabricate multilayer architectures, and its solvent-free nature.^31,32^ Huang et al. showed that an atmosphere controlled annealing process of vacuum deposited CsPbI_3_ perovskite film results in high PCE up to 16%.^33^ Nevertheless, the high temperature (350 °C) needed for fabrication limits the application of this method. Recently, Zhang et al. demonstrated stable and efficient thermal evaporated γ-CsPbI_3_ upon incorporation of phenethylammonium iodide (PEAI).^34,35^ The addition of PEAI during formation of the perovskite layer leads to preferred crystal orientation, improved microstructure, and reduced density of defects. In this work, we demonstrate the vacuum processing of CsPbI_3_ perovskite films at room temperature, obtained by incorporating DMAI by cosublimation with CsI and PbI_2_. As-prepared films were applied in planar solar cells, leading to an average PCE exceeding 12%. In order to improve the perovskite formulation and device performance, we introduced a third A-site cation (MA^+^) in a four-source deposition process. This prompted the formation of homogeneous films and efficient all-vacuum process wide bandgap solar cells with a PCE up to 14.8%.

The thin film deposition was carried out in a vacuum chamber equipped with four thermal sources, each with its own shutter and dedicated quartz crystal microbalance (QCM) thickness sensor. It is important to highlight that the perovskite layer was obtained at room temperature (RT), without an additional step of annealing. Initially, we tested the simultaneous cosublimation of CsI and PbI_2_ (deposition rates *r* of 0.6 Å/s and 1.2 Å/s, respectively) from two geometrically opposite thermal sources with respect to the sample holder (Figure 1a), which was kept fixed during the deposition. This allows us to create a compositional gradient and hence facilitate the direct observation of the eventual CsPbI_3_ formation.^36^ As clearly seen in Figure 1a, the substrates on the left side (closer to the CsI source) show a darker color, indicating the formation, to some extent, of CsPbI_3_ under CsI-rich conditions. On the right, PbI_2_-rich compositions do not lead to the formation of the perovskite phase, as the films appear yellow, suggesting the formation of the δ-CsPbI_3_ phase. This observation is expected and agrees with similar previous experiments carried out by Becker et al., where the γ-CsPbI_3_ perovskite phase was stabilized using CsI-rich formulations (and using a substrate temperature of 50 °C).^37^

**Figure 1:**
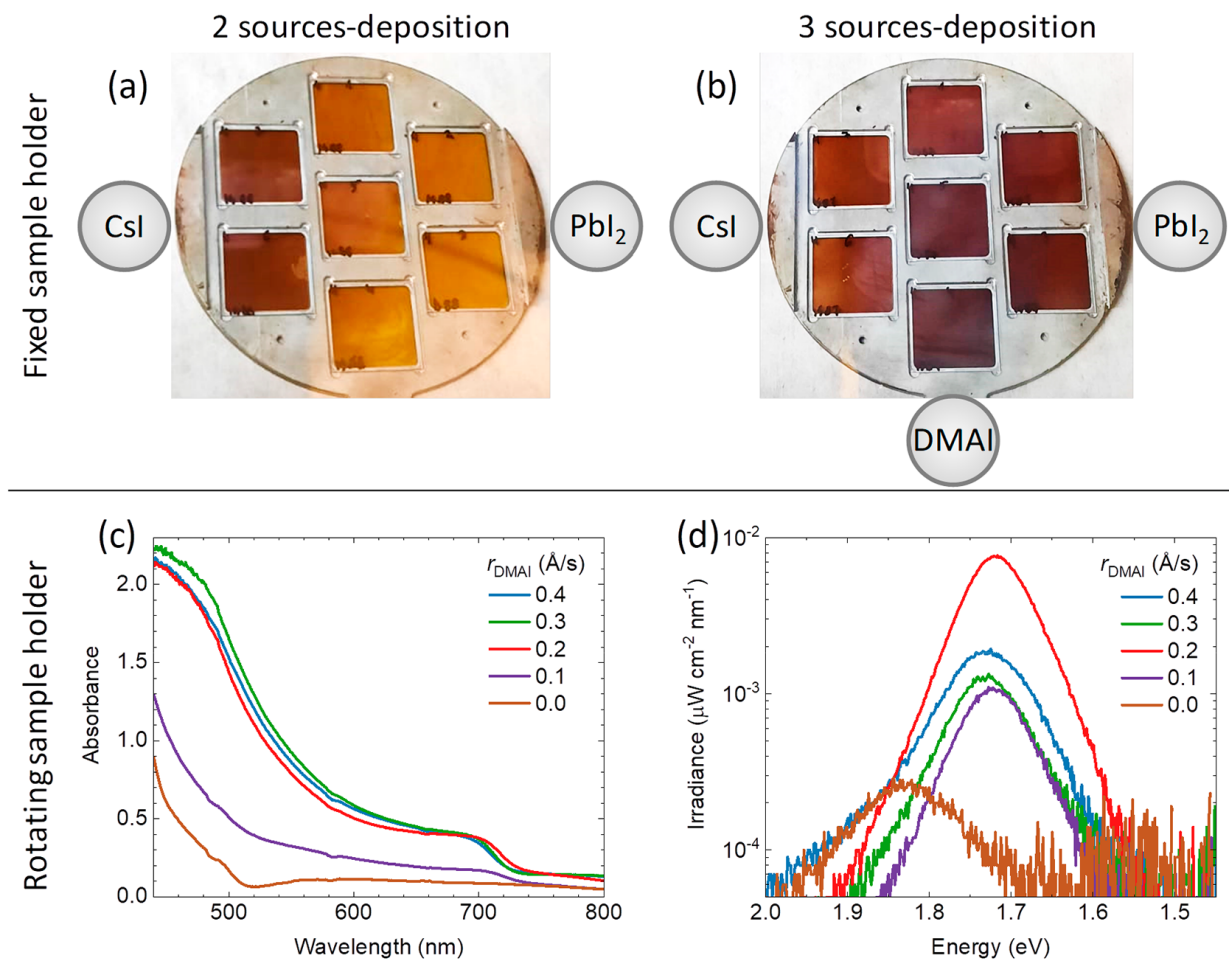
(a) Photograph of the sample holder with 7 as-deposited films after (a) 2- and (b) 3-source vacuum deposition without sample rotation. The position of the thermal sources with respect to the sample holder and the corresponding materials is also reported. (c) Optical absorption and (d) photoluminescence spectra of a series of CsDMAPbI_3_ perovskite films with varying DMA deposition rates, obtained with sample rotation.

In the attempt to obtain the γ-CsPbI_3_ perovskite at RT, we cosublimed also DMAI (*r*_DMAI_ = 0.3 Å/s) from a third source placed in the lower part of the sample holder, which is again fixed during the deposition. It is worth mentioning that despite the large size of the DMA^+^ cation, it can be sublimed in a high vacuum without any apparent decomposition, as observed by NMR (Figure S1). In the presence of DMAI, a dark CsPbI_3_ phase is observed in the lower and central substrates (Figure 1b), extending to the other surrounding substrates. As described in our previous work,^36^ the composition of the films in a deposition with substrate rotation resembles closely that of the central substrate when the sample holder is kept fixed. As the color of the central substrate in Figure 1b suggests the formation of the distorted γ-CsPbI_3_ perovskite at RT, we prepared a series of films with different amount of DMAI, this time with rotating sample holder. The absorbance spectra of 250 nm thick perovskite films with increasing *r*_DMAI_ are reported in Figure 1c. In absence of DMAI, the perovskite is not formed, as indicated by the absorption profile, which starts to raise only at approximately 520 nm (characteristic of the yellow δ-CsPbI_3_ phase). With *r*_DMAI_ = 0.1 Å/s, the absorbance is slightly increased and extends through the visible spectrum to approximately 720 nm. For higher *r*_DMAI_, as-deposited films show the expected perovskite absorption profile with an absorption cutoff at 700–720 nm and a strong rise in absorbance for wavelengths below 600 nm. The photoluminescence (PL) spectra of the same films, collected upon illumination with a 515 nm laser using an intensity that leads to a carrier concentration equal to what would be obtained under 1 sun illumination, are reported in Figure 1d. The film without DMAI shows a very weak (note the semilogarithmic scale) PL band centered at 1.83 eV maximum at 1.73 eV, which might be associated with the presence of small α-CsPbI_3_ domains in the yellow δ-CsPbI_3_ phase.^38^ When DMAI is incorporated in the perovskite, the PL signal is enhanced and centered to 1.72 eV (720 nm), in agreement with previous reports on the mixed A-cation Cs_*n*_DMA_1–*n*_PbI_3_ perovskite.^29^ This indicates that the cosublimation of DMAI with CsI and PbI_2_ leads to the formation of a mixed A-cation perovskite, rather than solely stabilizing γ-CsPbI_3_. The calibrated PL intensity (proportional to the PLQY) is observed to vary as a function of *r*_DMAI_, with a maximum for *r*_DMAI_ = 0.2 Å/s. The X-ray diffraction of the as-deposited films (Figure S2) shows a low signal-to-noise-ratio (SNR), suggesting a low degree of crystallization and/or the presence of amorphous material. While the low SNR precludes a detailed discussion of the structural properties of the films, the mixed compound Cs_*n*_DMA_1–*n*_PbI_3_ can be identified, together with a residual γ-CsPbI_3_ phase.

**Figure 2:**
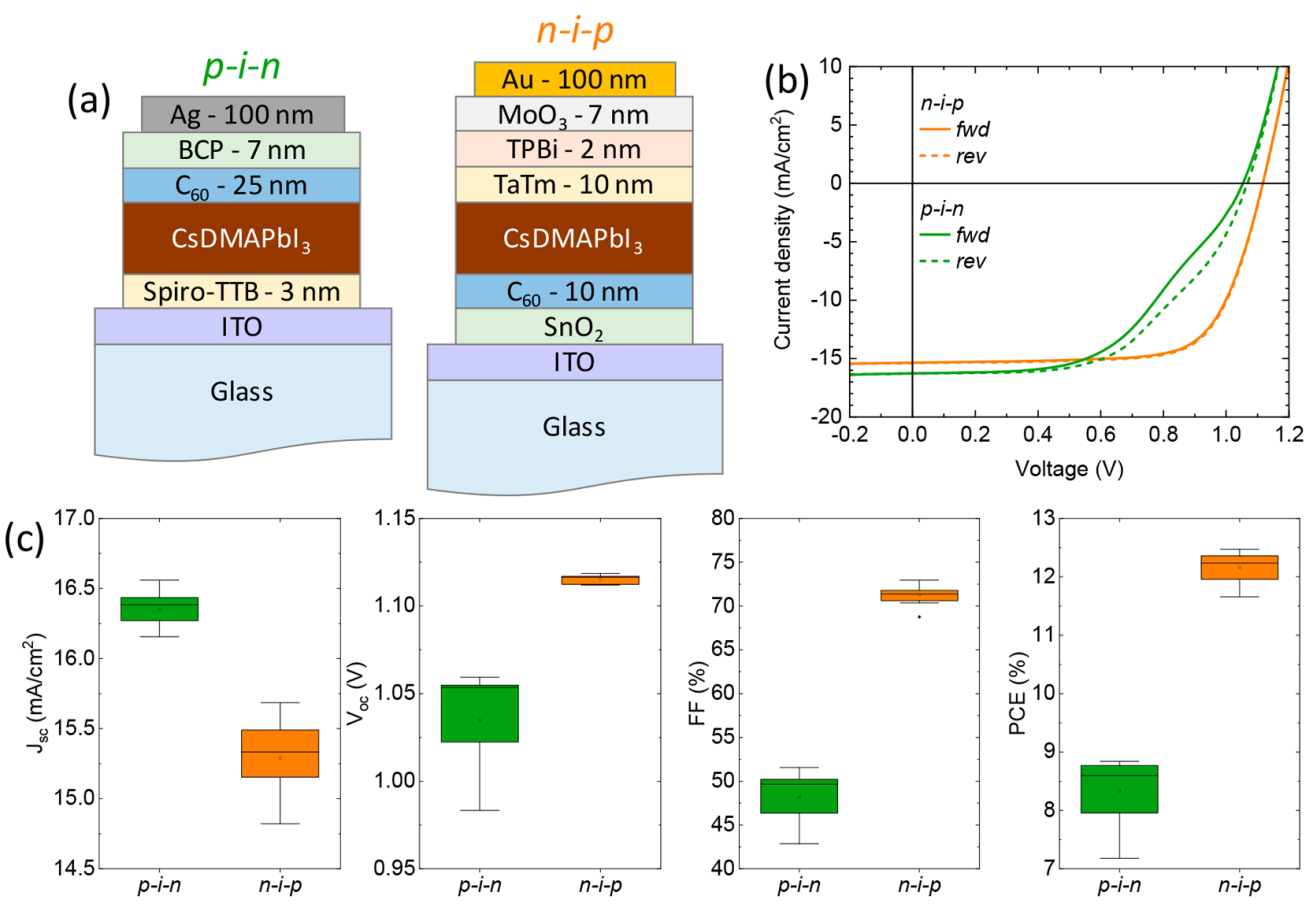
(a) Device layout for p-i-n and n-i-p cells used in this study. (b) Representative *J*–*V* curves for CsDMAPbI_3_ solar cells, obtained with *r*_DMA_ = 0.2 Å/s. The *J*–*V* curves are collected in forward (from short to open circuit, solid line) and reverse scan directions (from open to short circuit, dashed line). (c) PV parameters extracted from the same *J*–*V* curves.

In order to evaluate the potential of these materials, we used them in fully vacuum-processed solar cells, prepared in both the p-i-n and n-i-p configurations (Figure 2a). Glass substrates with patterned indium tin oxide (ITO) were used for device fabrication. The structure of p-i-n cells was: ITO/Spiro-TTB (3 nm)/Perovskite (250 nm)/C_60_ (25 nm)/BCP (8 nm)/Ag, where Spiro-TTB is 2,2′,7,7′-tetrakis(*N*,*N*′-di(*p*-methylphenyl)amino)-9,9′-spirobifluorene, C_60_ is fullerene, and BCP is bathocuproine. The inverted n-i-p cells had the structure: ITO/SnO_2_ (20 nm)/C_60_ (10 nm)/Perovskite (250 nm)/TaTm (10 nm)/TPBi (2 nm)/MoO_3_ (7 nm)/Au, where TaTm is N4,N4,N4″,N4″-tetra([1,1′- biphenyl]-4-yl)-[1,1′:4′,1″-terphenyl]-4,4″-diamine and TPBi is 2,2′,2″-(1,3,5-Benzinetriyl)-tris(1-phenyl-1-H-benzimidazole). SnO_2_ was deposited by atomic layer deposition (ALD),^39^ while MoO_3_ was thermally evaporated in a high vacuum. The fullerene layer was introduced in order to reduce carrier recombination at the SnO_2_ electron transport layer, as previously observed for vacuum deposited n-i-p solar cells on titanium oxide.^40,41^ All devices were subsequently encapsulated with a 20 nm thick Al_2_O_3_ film deposited with a low temperature ALD process.^42^ Details of the device fabrication are reported in the Experimental Section. Representative current-density versus voltage (*J*–*V*) curves (Figure 2b) under simulated solar illumination for p-i-n and n-i-p cells and statistics on the PV parameters (Figure 2c) for CsDMAPbI_3_ with *r*_DMA_ = 0.2 Å/s, are reported in Figure 2. The *J*–*V* curves for p-i-n and n-i-p devices differ substantially. For p-i-n cells, we observed a kink in the *J*–*V* curve, resulting in an average fill factor (FF) of 48%, as well as hysteresis between *J*–*V* scans from short to open circuit (forward, *fwd*) and open to short circuit (reverse, *rev*). Both observations are likely related to interface and/or bulk charge recombination,^43^ which limits also the open-circuit voltage (*V*_oc_) to 1.03 V, on average. In contrast, for n-i-p devices, we observed negligible hysteresis between forward and reverse scans, indicating that either ion migration or interface recombination (or both) are suppressed in this configuration.^44,45^ The *V*_oc_ in n-i-p cells is 1.12 V on average, with FF > 70% and short circuit current density (*J*_sc_) of 15.3 mA/cm^2^. Note that *J*_sc_ is higher for pin cells (16.4 mA/cm^2^ on average), due to the absence for the highly absorbing C_60_ film in the front part of the device. However, the resulting PCE for p-i-n cells was only slightly above 8%, while n-i-p cells delivered a promising average PCE of 12.1%. The reason for the superior performance of n-i-p solar cells is likely related to a higher diffusion length for holes as compared to electrons, which is supported by measurements on single-carrier devices (Figure S3). On the other hand, we cannot exclude the presence of a different electronic energy level alignment for the p-i-n and n-i-p cells, as the transport materials and buffer layers are not identical, which would change the built-in potential and hence results in a kink of the *J*–*V* curve, as observed in Figure 2b.

With the aim to improve the properties of the CsDMAPbI_3_ perovskite, we introduced a third A-cation, in particular MA^+^ or FA^+^. This led to a four-source deposition process, subliming simultaneously CsI, PbI_2_, DMAI, and MAI or FAI. As the intention is to introduce the A-cation as an additive, we kept its deposition rate low to 0.1 Å/s. The optical absorption of the triple-cation CsMADMAPbI_3_ and CsFADMAPbI_3_ films (Figure 3a) confirms the formation of perovskite in both cases, showing high absorbance below 600 nm and an absorption edge at approximately 700–720 and 720–740 nm when MAI and FAI are added, respectively. Perovskite films with incorporation of MAI exhibit a PL maximum (Figure 3b) at 1.73 eV (717 nm), whereas the perovskite films prepared with the addition of FAI show the PL maximum at 1.70 eV (729 nm).

**Figure 3:**
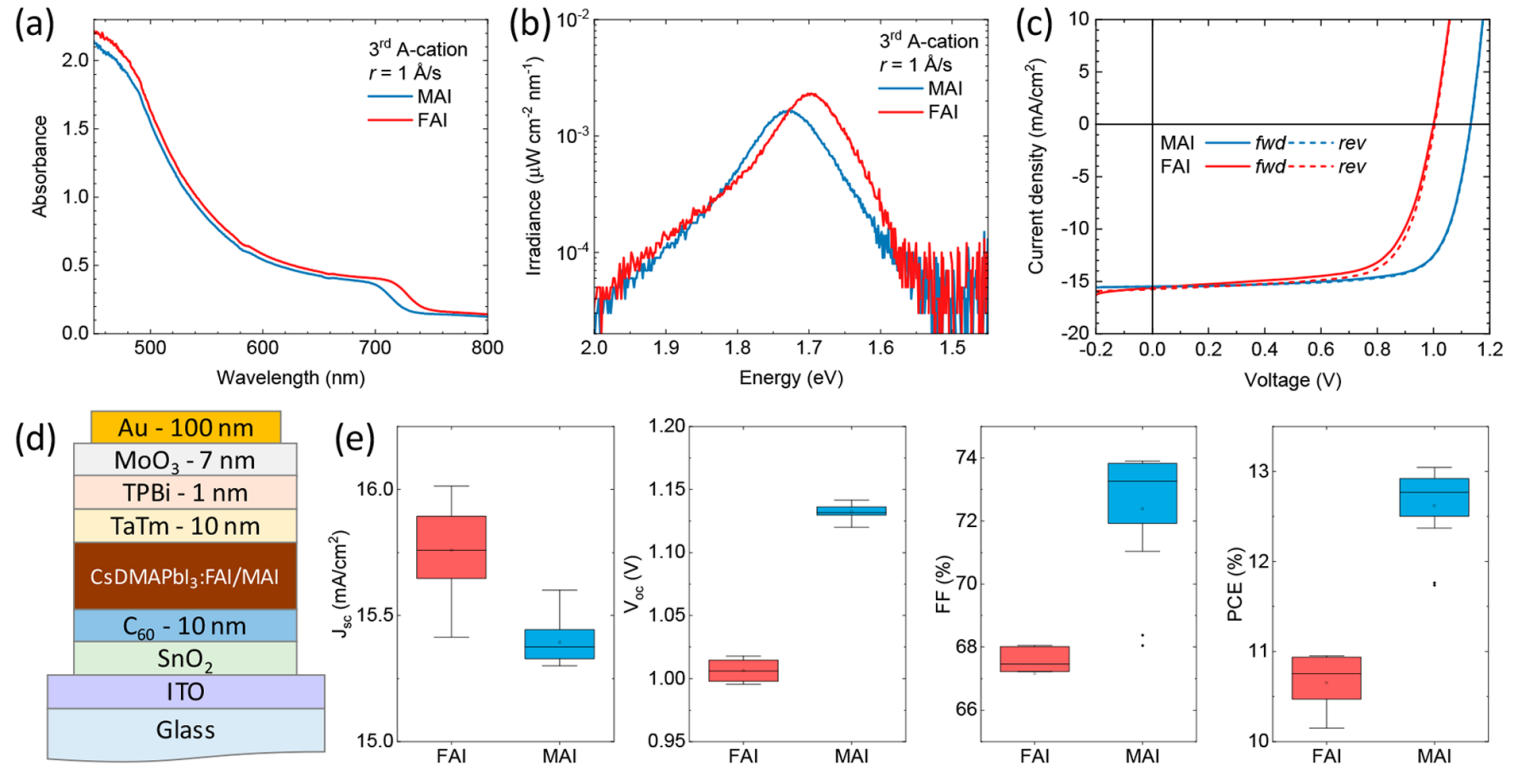
(a) Absorption and (b) PL spectra for triple-cation perovskites obtained by adding MAI (blue) or FAI (red) to the previously developed CsDMAPbI_3_ perovskite, using a fourth source and *r* = 0.1 Å/s. (a) *J*–*V* curves under illumination for representative MA_DMACsPbI_3_ and FA_DMACsPbI_3_ solar cells and corresponding (b) PV parameters extracted from *J*–*V* curves.

From the above, the addition of MAI results in a slightly blue-shifted PL (wider bandgap) as compared to the reference CsDMAPbI_3_ perovskite, while a narrower bandgap is observed upon addition of FAI. In a simplified view, this observation can be explained by the impact of the ionic radii of the additional cations: being smaller (217 pm), MA^+^ would reduce the perovskite tolerance factor, slightly widening the bandgap, while FA^+^ (253 pm) would have the opposite effect.^46^ The triple-cation perovskite films were tested in n-i-p devices with the structure reported in Figure 3d. The solar cells based on the perovskites obtained with FAI showed lower performance, reaching an average PCE of 10.7%. This is related to the low *V*_oc_ and FF (about 1 V and 67%, respectively). The solar cells prepared with CsMADMAPbI_3_ results in slightly improved PCE, up to 13%, as compared to the CsDMAPbI_3_ based devices shown in Figure 2, a consequence of a small increase of the *V*_oc_ and FF (1.13 V and 73%, on average). The *J*_sc_ was found to be 15.4 mA/cm^2^ on average, likely a consequence of the thin absorber layer used. From the EQE spectrum in Figure S4, one can notice a strong reduction of the EQE in the low energy region, which is a signature of insufficient thickness of the perovskite layer.^47^ Hence we fabricated solar cells with increased thickness of perovskite layer (400 nm) to try to compensate for the current density loss.

The *J*–*V* curves of these devices (Figure 4a) did not show any appreciable hysteresis, closely resembling the ones with the thinner perovskite absorber (Figure 3c), but the current density was found to be increased to 17 mA/cm^2^. This is a consequence of the enhanced spectral response in the red part of the visible spectrum. A comparison of the EQE and absorption spectra, as well as the first derivative of the EQE (showing an effective *E*_g_ = 1.70 eV) are reported in Figure S5. The *V*_oc_ and FF were almost unaltered (about 1.15 V and 74%), resulting in a very promising PCE of 14.4% on average and with record pixels with PCE of 14.8% (the corresponding PV parameters are *J*_sc_ = 17.2 mA/cm^2^; *V*_oc_ = 1.157 V; FF = 74.6%). This value is on par with the best reported, pure iodide wide bandgap perovskite solar cells^34^ prepared by thermal evaporation. It is worth noticing that the photocurrent could be increased by further increasing the perovskite thickness, as suggested by the still not-optimum response in the low energy region of the EQE. This is certainly true when testing solar cells with 600 nm thick perovskite films; however, the other parameters were found to decrease, reducing the overall PCE (Figure S6). We also tested the effect of MAI on the stability of the perovskite, comparing the optical properties (absorption and PL) of CsDMAPbI_3_ and CsMADMAPbI_3_ films stressed under 1 sun illumination, at 85 °C, and under illumination at 85 °C (Figure S7). For both compounds we observed a good stability toward light soaking (over the course of 2 days), but the MA-substituted perovskite was shown to be more stable when stressed at 85 °C. A similar trend is also observed when the perovskite films are tested at 85 °C under illumination, where, however, a faster degradation is observed for both materials. From these observations, it seems that MA is capable of partially stabilizing the pure iodide, wide bandgap perovskite phase, although the overall stability of the samples at high temperature and under illumination is still limited.

**Figure 4:**
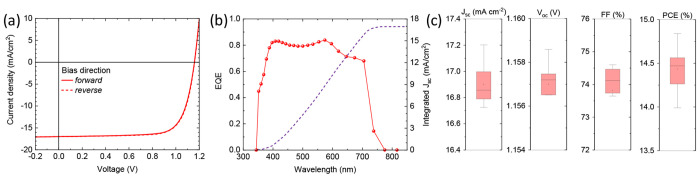
(a) *J*–*V* curves for a triple-cation CsMADMAPbI_3_ perovskite solar cell with a 400 nm thick absorber layer. (b) EQE spectrum with integrated current density and (c) PV parameters extracted from *J*–*V* curves.

Among the major benefits of thermal evaporation is the wide substrate compatibility, for example, allowing the deposition of perovskite films on both flat and textured surfaces. Here we show a conformal coating of the triple-cation CsMADMAPbI_3_ perovskite on top of textured silicon substrate with 3–5 μm pyramidal height. The cross-section SEM shows a conformal coating of the textured silicon with the CsMADMAPbI_3_ perovskite (Figure 5a). Importantly, the perovskite film is uniform in terms of morphology and thickness and appears very compact with low porosity. Therefore, this perovskite composition is also promising for perovskite/silicon tandem devices, where complete and conformal coverage is required.

**Figure 5:**
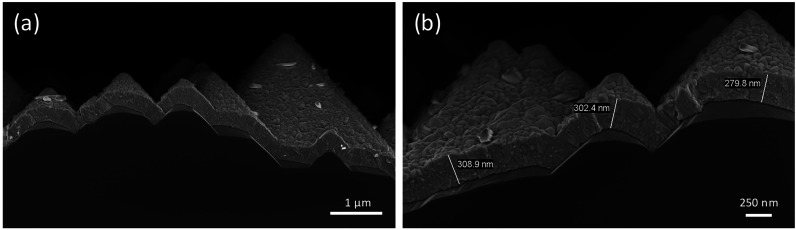
(a) Cross-sectional SEM of a CsMADMAPbI_3_ perovskite film deposited on a textured silicon substrate. (b) Zoom of the same sample highlighting the uniform thickness and low porosity of the perovskite coating.

In summary, we investigated the vacuum deposition of pure iodide wide bandgap perovskites at room temperature. CsPbI_3_ deposition at RT results predominantly in the formation of the yellow δ-phase, as previously reported. The black phase can be stabilized with the incorporation of a larger cation, DMAI, allowing the formation of a mixed CsDMAPbI_3_ perovskite at RT. We have investigated the optoelectronic properties of this material in planar p-i-n and n-i-p solar cells, with the latter exhibiting superior performance, likely due to a non ambipolar charge transport in the material. We have further studied the use of a third A-site cation, adding formamidinium (FA^+^) and methylammonium (MA^+^) in a four-source vacuum deposition process. The use of MAI resulted in more favorable optoelectronic properties, and by tuning the perovskite thickness, promising efficiencies of up to 14.8% were obtained for pure iodide, wide bandgap perovskite solar cells completely prepared by vacuum/vapor deposition methods. This triple-cation perovskite can be coated on top of the textured silicon used in Si solar cells, making the material and process promising for perovskite/silicon tandem solar cells. Future studies will target methodologies to control the degree of crystallization of vacuum deposited perovskite films, whose structure and morphology are still inferior to their solution-processed counterparts. We believe that this will benefit both performance and stability, as structural defects are nonradiative recombination centers and are likely initiators of the material degradation.

## Supporting Information Available

Experimental section, ^1^H NMR analysis of precursors and films, single carrier devices, XRD pattern, schematics of the planar single-carrier devices, band energy diagrams, EQE measurements, *J*–*V* curve, PV paramaters, light soaking and thermal stability analysis; table of summary of the EDS analysis; ^1^H NMR spectrum of perovskite films (PDF)
