# Supplementary material for: Pure Iodide Multication Wide Bandgap Perovskites by Vacuum Deposition
Source: ACS Mater Lett. 2023 Nov 13;5(12):3299–305. doi: 10.1021/acsmaterialslett.3c01094 (PMC10851662; doi:10.1021/acsmaterialslett.3c01094)
Supplement: Supplementary file 1 — tz3c01094_si_001.pdf [file tz3c01094_si_001.pdf]

Supporting Information

Pure Iodide Multication Wide Bandgap Perovskites by Vacuum Deposition

Isidora Susic[1], Lidón Gil-Escrig[1], Kassio P. S. Zanoni[1], Cristina Roldán-Carmona[1], Michele Sessolo[1] and Henk J. Bolink[1]*

[1]Instituto de Ciencia Molecular, Universidad de Valencia, C/Catedrático J. Beltrán 2, Paterna, 46980 Spain

Experimental section

Materials. TaTm and CsI were purchased from Tokyo Chemical Industry CO (TCI), Fullerene (C_{60}) was purchased from Merck KGaA. PbI_2, MAI, MoO_3, Spiro-TTB, TPBi, BCP were purchased from Luminescence Technology Corp. DMAI and FAI were purchased from Greatcell Solar. All materials were used as received.

Thin film and device preparation. ITO-coated glass substrates were subsequently cleaned with soap, water and isopropanol in an ultrasonic bath, followed by 20 min UV-ozone treatment. All further processing was carried out in nitrogen-filled gloveboxes. For n-i-p configuration, a20 nm layer of SnO_2 was deposited by ALD using an Arradiance's GEMStar XT Thermal ALD system integrated into a nitrogen-filled glovebox. 1 For that, the ALD chamber was heated to 90 °C; one ALD cycle consisted of consecutive purges of tetrakis(diethylamino)tin (TDAT) for 550 ms and water vapor for 200 ms, each followed by N_2 purges of 30 and 105 s, respectively (final growth per cycle: 1.5 Å).C_{60} was deposited in another vacuum chamber with deposition rate of 0.5 Å s^{-1}. The substrates were transferred to a vacuum chamber integrated in a nitrogen-filled glovebox and evacuated to a pressure of 10^{-6} mbar for the perovskite deposition. The chamber is equipped with four evaporation sources (Creaphys) and with independent temperature controllers and shutters. All sources have a dedicated quartz crystal microbalance (QCM) sensor above, and an additional

one is installed close to the substrates for the overall deposition rate measurement. All sources were individually calibrated for their respective materials and no cross-reading between the different QCMs is ensured by the relative position of the sources, shutters, sensors. For thickness calibration, we individually sublimed each material, and a calibration factor was obtained by comparing the thickness inferred from the QCM sensors with that measured with a mechanical profilometer (Ambios XP1). During the perovskite deposition, the pressure of the chamber was maintained at $8\cdot10^{-6}$ mbar and the substrates were kept at room temperature. Typical sublimation temperatures for the precursors were approximately 170 ºC for DMAI (r = 0.2 Å s^{-1}), 310 ºC for PbI_2 (r = 1.2 Å s^{-1}), 520 ºC for CsI (r = 0.6 Å s^{-1}), 100 ºC for MAI or FAI (r = 0.1 Å s^{-1}). Charge extraction layers were deposited in a second chamber integrated in a nitrogen-filled glovebox. The deposition rate for TaTm was 0.5 Å s^{-1} and for MoO_3 0.1 Å s^{-1}. Au was deposited in a third vacuum chamber from alumina-coated aluminum boats, and by applying currents ranging from 2.0 to 4.5 A. For p-i-n configuration, Spiro-TTB was sublimed with a deposition rate of 0.1 Å s^{-1}. After deposition of a perovskite film, the layer of C_{60} (25 nm) and a thin layer of BCP (7 nm) were evaporated at a rate of 0.5 and 0.3 Å s^{-1}, respectively. To finish the device, the metal top contact Ag (100 nm) was sublimed in a third vacuum chamber. All devices were encapsulated by ALD coating, a Arradiance's GEMStar XT Thermal ALD system integrated into a nitrogen-filled glovebox was used. The ALD chamber was heated to 40 ºC, while the precursor and oxidizer manifolds (to which the bottles of trimethylaluminum (TMA) and water were connected) were heated to 115 and 140 ºC, respectively, to avoid vapor accumulation at the tubes leading to the main chamber. Prior to deposition, the tubes and valves were degassed three times by performing pulses with the bottles manually closed, to avoid contamination. The substrates were inserted in the chamber, which was then evacuated. A N_2 gas flow of 20 SCCP was used as TMA and water vapor carrier. A cycle consisted of consecutive purges of TMA for 10 ms and water vapor for 30 ms, each followed by N_2 purges for enough time to guarantee complete removal of the precursors from the ALD chamber, as monitored by the transient pressure inside the chamber.

Thin film and device characterization. Absorption spectra were collected using fiber optics based Avantes Avaspec2048 Spectrometer. The photoluminescence spectra were measured with an Avantes Avaspec2048 spectrometer and films were illuminated with a diode laser of Integrated

Optics, emitting at 515 nm. All the spectra were collected with an integration time of 1 s. The crystalline structure of the thin films was studied by X-ray diffraction (XRD). The patterns were collected in Bragg-Brentano geometry on an Empyrean PANalytical powder diffractometer with a copper anode operated at 45 kV and 40 mA. Scanning Electron Microscopy (SEM) images were performed on a Hitachi S-4800 microscope operating at an accelerating voltage of 2 kV over platinum-metallized samples. The J–V curves for the solar cells were recorded using a Keithley 2612A SourceMeter in a 0.2 and 1.2 V voltage range, with 0.01 V steps and integrating the signal for 20 ms after a 10 ms delay, corresponding to a speed of about 0.3 V s^{-1}. The devices were illuminated under a Wavelabs Sinus 70 LED solar simulator. The light intensity was calibrated before every measurement using a calibrated Si reference diode.

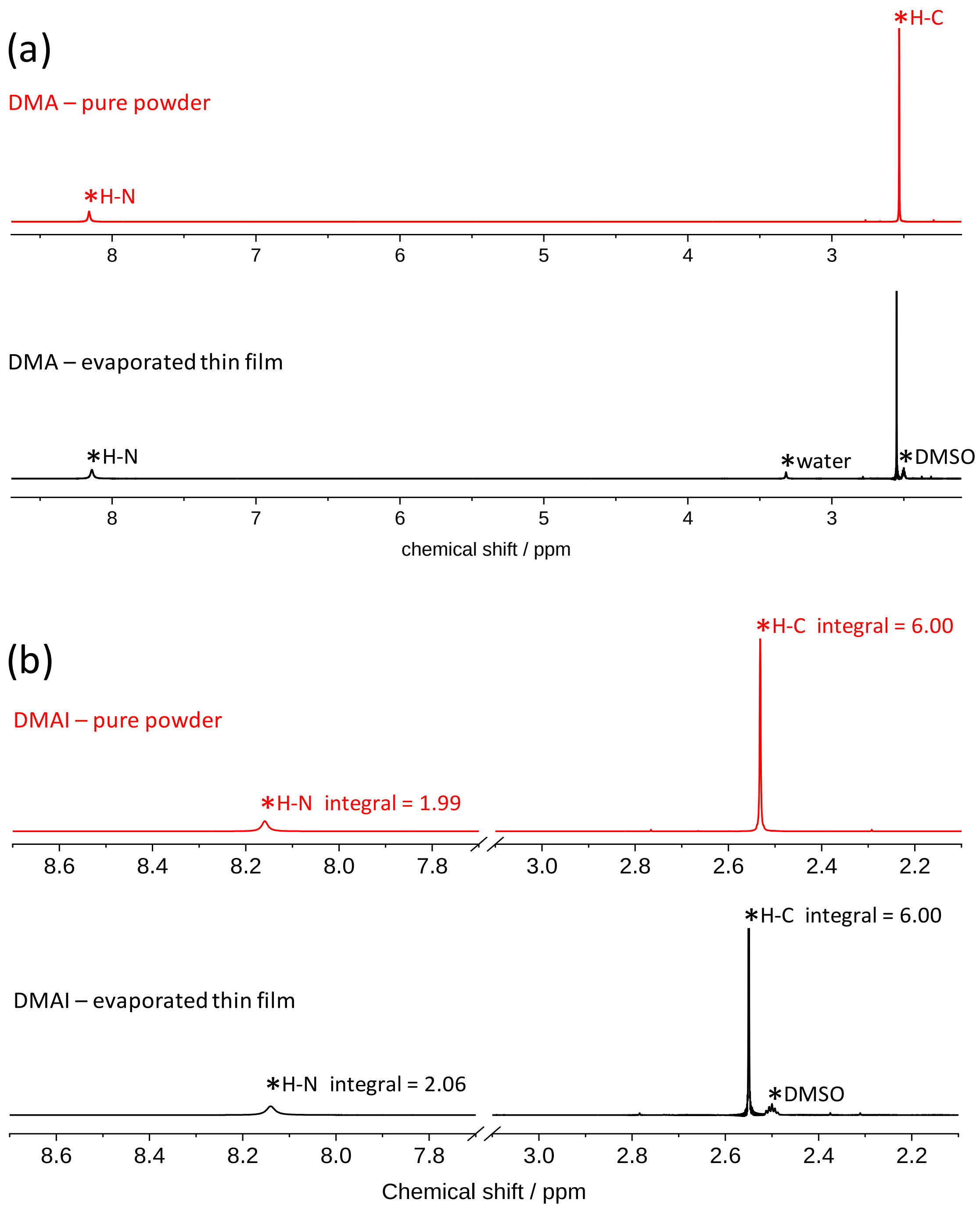

Figure S1. ^{1}H-NMR spectra of the commercial reference DMAI (red) and of the vacuum deposited DMAI films, both dissolved in deuterated DMSO. In (a) the wide spectra to show that there are no other secondary products during evaporation and (b) zoom spectra on H-N and H-C protons signals with corresponding integration, confirming in both cases the stoichiometry $(CH_3)_2NH_2^+$.

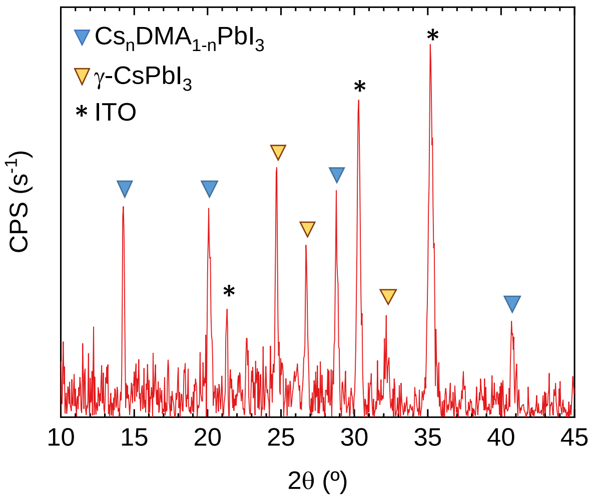

Figure S2. XRD pattern of a thin perovskite film deposited on ITO, obtained by co-sublimation of PbI_2 (1.2 Å/s), CsI (0.6 Å/s) and DMAI (0.2 Å/s). The main observable phases are assigned following literature reports.[2,3]

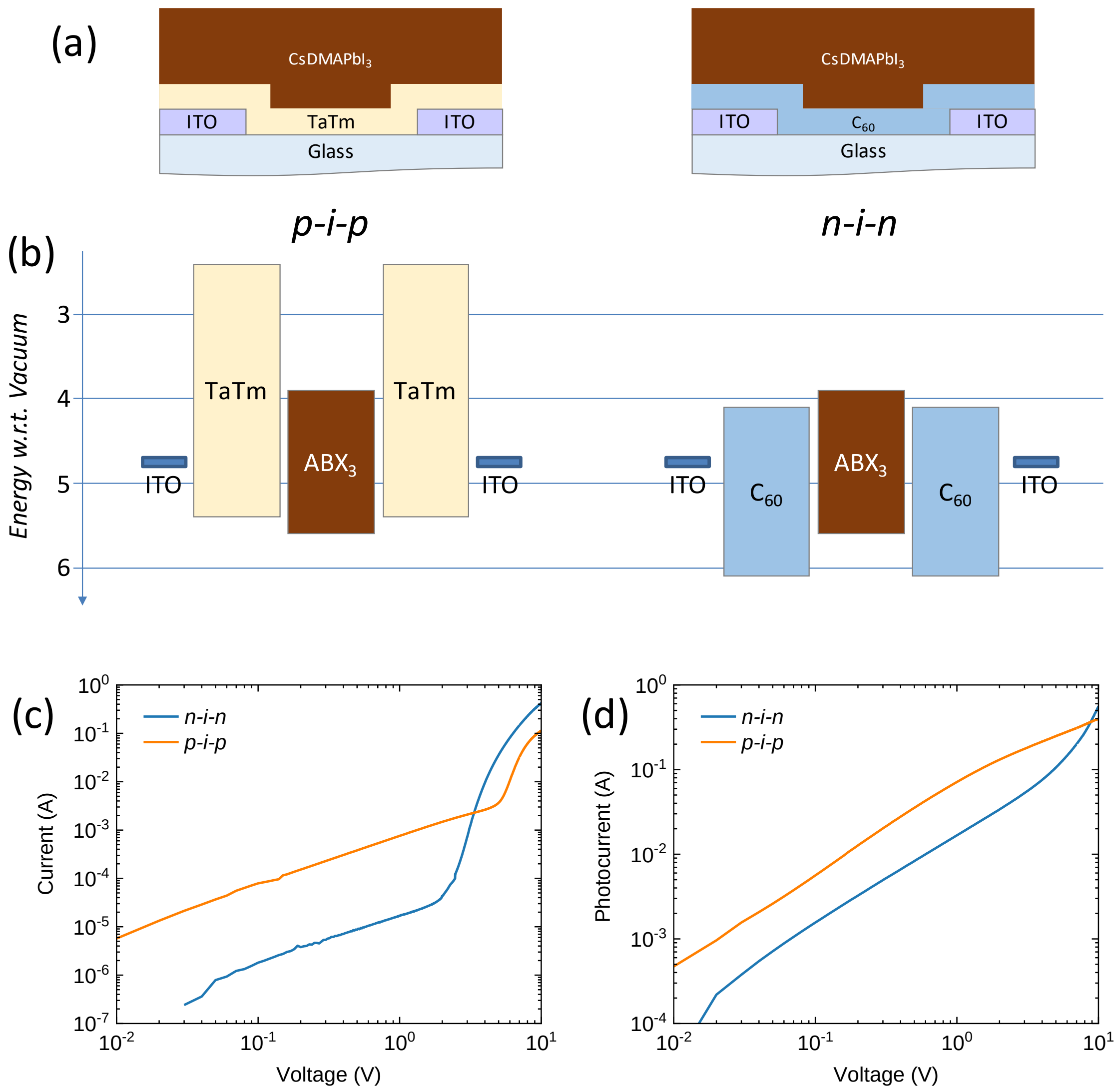

Figure S3. (a) Schematics of the planar single-carrier devices obtained by vacuum deposition of the perovskite films on interdigitated ITO electrodes. The perovskite and transport layer thickness are the same as in the solar cells. (b) Flat band energy diagrams for the materials used in the single carrier devices, p-i-p (hole-only devices) and n-i-n (electron-only devices). J-V curves recorded (c) in the dark and (b) under illumination for the same samples.

Single carrier devices were prepared using the architecture showed in Figure S3a. The devices are fabricated on interdigitated ITO electrodes (with inter-electrode spacing of 40 μm) and coated with a selective transport layer, below the perovskite film. In this way, symmetric devices (schematic flat energy band diagram shown in Figure S3b) are obtained. At low applied electric field and dark

conditions, the current is dominated by a single carrier (holes in p-i-p and electrons in n-i-n), while at higher bias the carriers with opposite charge can also be injected.

As it can be observed in Figure S3c, the dark current at low bias for p-i-p devices is almost two orders of magnitude higher than in n-i-n devices, suggesting that the $CsDMAPbI_3$ perovskite is more p-type. At higher bias, we see injections of the opposite carrier type, and the current becomes larger for n-i-n devices: this is because the barrier for hole injection in n-i-n devices (ITO to C60 HOMO) is smaller as compared to the barrier for electron injection in p-i-p cells (ITO to TaTm LUMO). Under illumination (Figure S3d), the devices behave as photoconductors, where the steady-state photoconductivity σ_{ph} can be simplified as:

$$\sigma_{ph} = eG(\mu\tau)_{maj}$$

Where e is the elementary charge, G the photogeneration rate, $(\mu\tau)_{maj}$ the mobility -lifetime product for the majority carriers.[1] Also in this case, assuming that the generation rate is the same and that at low bias only one of the carrier can circulate in the circuit, we observe a larger photocurrent (close to one order of magnitude) for the p-i-p devices, indicating that the $\mu\tau$ product is higher in the case of holes. These data would support the assumption that the diffusion length is higher for holes as compared to electrons in this particular perovskite.

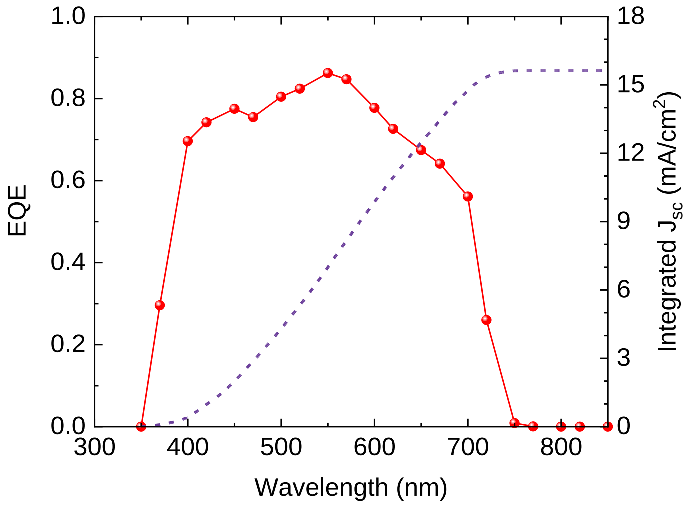

Figure S4. EQE spectrum (red) of a CsMADMAPbI_3 perovskite solar cell in the n-i-p configuration, with 250 nm thick perovskite, and corresponding integrated short-circuit current density with the AM1.5G solar irradiance spectrum.

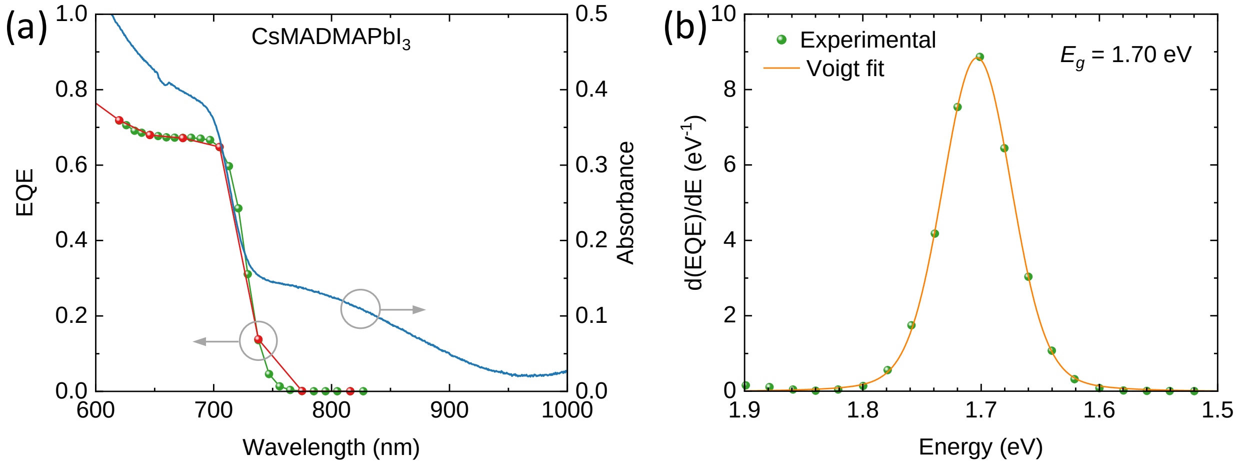

Figure S5. (a) Comparison of low (red line with symbols) and high (green line with symbols) resolution EQE spectra (left) with the absorption spectra (right, blue line) collected from 400 nm thick CsMADMAPbI_3 perovskite device and film. (b) First derivative of the EQE spectrum in the bandgap region fitted with a Voigt function to identify the effective bandgap.

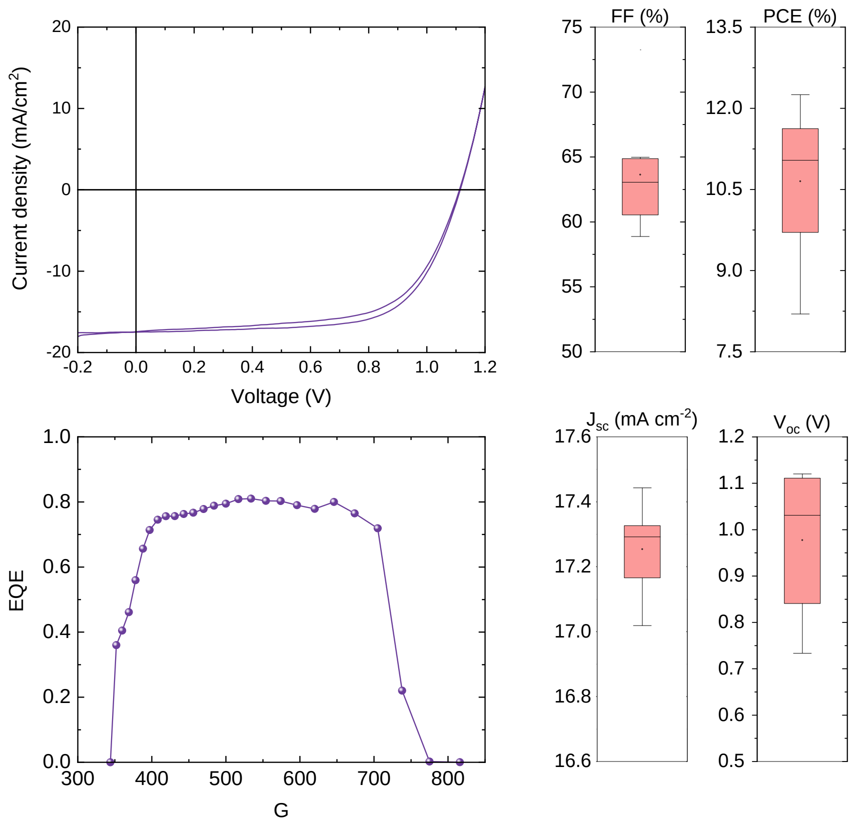

Figure S6. J-V curve, EQE spectrum and PV parameters for solar cells obtained with a 600 nm thick $CsMADMAPbI_3$ perovskite absorber.

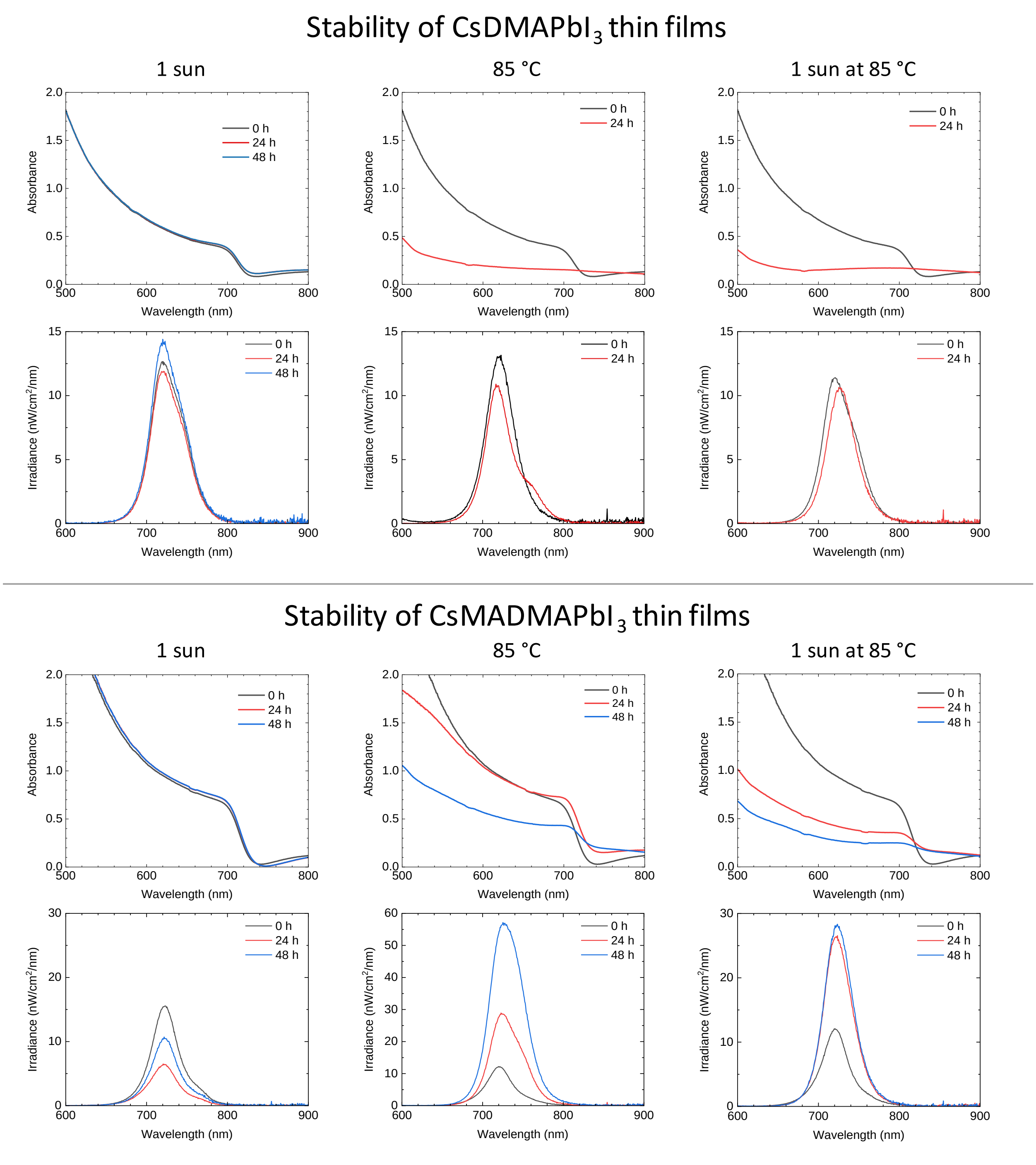

Figure S7. Comparison of the stability, assessed by optical absorption and photoluminescence, of the CsDMAPbI3 perovskite (top) with the MA-substituted $CsMADMAPbI_3$ perovskite. Films were stressed under 1 sun equivalent illumination (left), at 85 °C in the dark, and under 1 sun illumination at 85 °C.

Table S1: Summary of the EDS analysis carried out on two $CsMADMAPbI_3$ perovskite films, on different regions for each sample to check for homogeneity.

	Sample 1, normalized at%			Sample 2, normalized at%				
Element	Region 1	Region 2	Region 3	Region 1	Region 2	Region 3	Average	Ratio to Pb
Cs	8.96	10.78	8.76	9.96	10.86	9.71	9.8	**0.40**
Pb	24.08	25.77	25.29	23.65	23.20	24.10	24.3	**1.00**
I	66.95	63.45	65.94	66.39	65.94	66.19	65.8	**2.71**

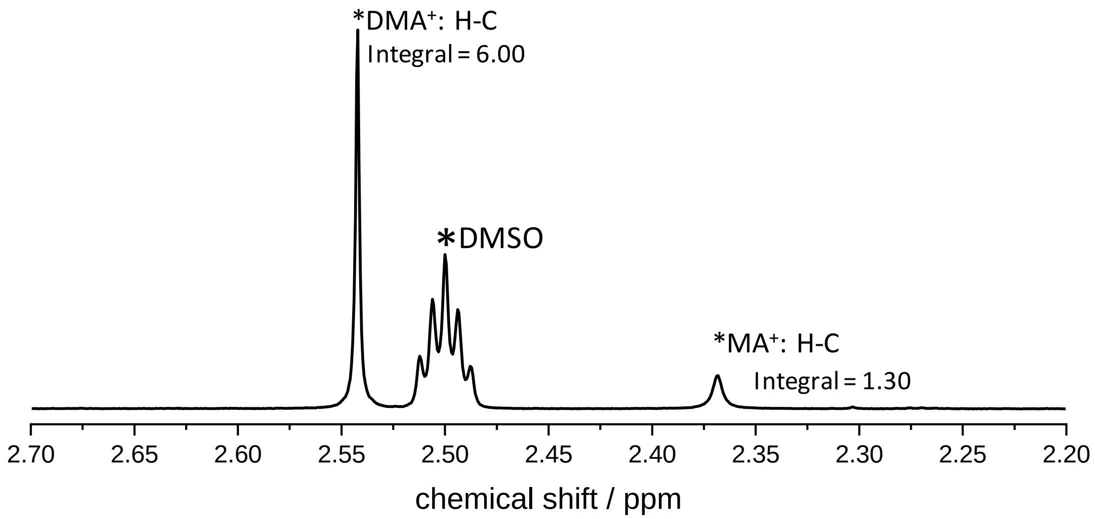

Figure S8. 1H-NMR spectrum of $CsMADMAPbI_3$ perovskite films dissolved in deuterated DMSO. The proton signals corresponding to the methyl groups of dimethylammonium and methylammonium can be clearly observed, and from their integration we can estimate a DMA^+/MA^+ molar ratio of 1.00/0.43. Considering the EDS analysis reported in Table S1, we can estimate a perovskite composition of $Cs_{0.4}MA_{0.2}DMA_{0.4}PbI_3$.

References

1. Brüggemann, R. Steady-state photocarrier grating technique for the minority-carrier characterisation of thin-film semiconductors. *J. Phys. Conf. Ser.* **253**, 012081 (2010).

2. Ke, W., Spanopoulos, I., Stoumpos, C. C. & Kanatzidis, M. G. Myths and reality of HPbI3 in halide perovskite solar cells. *Nat. Commun.* **9**, 4785 (2018).

3. Becker, P. *et al.* Low Temperature Synthesis of Stable γ-CsPbI 3 Perovskite Layers for Solar Cells Obtained by High Throughput Experimentation. *Adv. Energy Mater.* **9**, 1900555 (2019).
